# Neuronal Culture Microenvironments Determine Preferences in Bioenergetic Pathway Use

**DOI:** 10.3389/fnmol.2017.00305

**Published:** 2017-09-29

**Authors:** Juliane Sünwoldt, Bert Bosche, Andreas Meisel, Philipp Mergenthaler

**Affiliations:** ^1^Charité – Universitätsmedizin Berlin, Department of Experimental Neurology, Berlin, Germany; ^2^Division of Neurosurgery, Keenan Research Centre for Biomedical Science and the Li Ka Shing Knowledge Institute, St. Michael’s Hospital, University of Toronto, Toronto, ON, Canada; ^3^Department of Neurology, University Hospital of Essen, University of Duisburg-Essen, Essen, Germany; ^4^Institute of Neurophysiology, Medical Faculty, University of Cologne, Cologne, Germany; ^5^Department of Neurocritical Care, First Stage Rehabilitation and Weaning, MediClin Klinik Reichshof, Eckenhagen, Germany; ^6^Charité – Universitätsmedizin Berlin, Department of Neurology, Berlin, Germany; ^7^Charité – Universitätsmedizin Berlin, Center for Stroke Research Berlin, Berlin, Germany; ^8^Charité – Universitätsmedizin Berlin, NeuroCure Clinical Research Center, Berlin, Germany; ^9^Berlin Institute of Health (BIH), Berlin, Germany

**Keywords:** disease modeling, energy metabolism, glycolysis, metabolic flux analysis, neuronal energy metabolism, neuronal survival, oxidative phosphorylation, cell culture microenvironment

## Abstract

In the brain, metabolic supply and demand is directly coupled to neuronal activation. Methods for culturing primary rodent brain cells have come of age and are geared toward sophisticated modeling of human brain physiology and pathology. However, the impact of the culture microenvironment on neuronal function is rarely considered. Therefore, we investigated the role of different neuronal culture supplements for neuronal survival and metabolic activity in a model of metabolic deprivation of neurons using oxygen deprivation, glucose deprivation, as well as live cell metabolic flux analysis. We demonstrate the impact of neuronal culture conditions on metabolic function and neuronal survival under conditions of metabolic stress. In particular, we find that the common neuronal cell culture supplement B27 protects neurons from cell death under hypoxic conditions and inhibits glycolysis. Furthermore, we present data that B27 as well as the alternative neuronal culture supplement N2 restrict neuronal glucose metabolism. On the contrary, we find that the more modern supplement GS21 promotes neuronal energy metabolism. Our data support the notion that careful control of the metabolic environment is an essential component in modeling brain function and the cellular and molecular pathophysiology of brain disease in culture.

## Introduction

In the brain, neuronal function is directly coupled to metabolic activity, and neuronal computation regulates metabolic supply and demand (Dohmen et al., 2003; Bosche et al., 2010; Mergenthaler et al., 2013; Magistretti and Allaman, 2015). Indeed, the brain uses most of its energy consumption to sustain synaptic activity under physiological conditions (Alle et al., 2009; Harris et al., 2012; Mergenthaler et al., 2013). In addition to a central role for the pathophysiology of epileptic seizures (Arsov et al., 2012; Lutas and Yellen, 2013) or cortical spreading depressions (Dohmen et al., 2008; Bosche et al., 2010; Feuerstein et al., 2016), glucose metabolism, metabolic deprivation, and disturbed metabolic pathways are emerging as important pathophysiological mechanisms in acute (Mergenthaler et al., 2012; Quaegebeur et al., 2016) and chronic (Funfschilling et al., 2012; Mergenthaler et al., 2013) neurodegeneration and cell death mechanisms in the brain. Glucose metabolism and cell death regulation meet at mitochondria (King and Gottlieb, 2009) and glucose-metabolizing enzymes have been shown to be involved in cell death regulation under various circumstances (Buchakjian and Kornbluth, 2010; Mergenthaler et al., 2012, 2013; Andersen and Kornbluth, 2013) as well as apoptosis-regulating proteins to influence glucose metabolism (Gimenez-Cassina and Danial, 2015).

Methods for the cultivation of rodent primary neurons and other brain cells have been established for decades (Bottenstein and Sato, 1979; Brewer et al., 1993). Given the fundamental role of cellular model systems for biological discovery and investigating molecular and cellular mechanisms in brain function, and the role the extracellular environment plays in determining intracellular function (Bosche et al., 2016; Guo et al., 2016), we here investigated the role of the culture microenvironment on the preferential use of distinct metabolic pathways in neurons.

## Materials and Methods

### Primary Neuronal Cultures

Wistar rats were handled in accordance with institutional guidelines and with permission of the *Landesamt für Gesundheit und Soziales (LAGeSo), Berlin*. Brains of day 17 Wistar rat embryos (E17) were isolated and cortices were dissected and seeded with a density of 175,000 cells per cm^2^ and cultured for 9 days as described (Mergenthaler et al., 2012) unless otherwise stated. Briefly, primary cortical neurons were seeded in Neurobasal medium (NBM; Invitrogen, Thermo Fisher Scientific) supplemented with 25 μM glutamate, 0.5 mM L-glutamine, and a serum-free supplement (B27, N2, Invitrogen, Thermo Fisher Scientific, or GS21, MTI-GlobalStem). B27, N2, and GS21 are based on published formulations (Bottenstein and Sato, 1979; Brewer et al., 1993; Chen et al., 2008); see **Table 1** for details on their composition. The medium was partially replaced every 4 days with NBM supplemented with 0.5 mM L-glutamine and serum-free supplement. Alternatively, neurons were cultured in BrainPhys medium instead of NBM which was prepared in house based on the published formulation (Bardy et al., 2015) prior to its commercial availability (see **Table 2** for the exact formulation used herein). The bicarbonate concentration was slightly decreased to 26 mM to adjust the pH to 7.40. Primary cortical neurons were seeded in BrainPhys supplemented with 25 μM glutamate and a serum-free neuronal supplement (B27, N2, or GS21), and the medium was partially replaced during the cultivation period as described above.

**Table 1:** Formulation of the B27, GS21, and N2 supplements.

Component	Concentration (μM)			
	B27 (B18)		GS21/NS21	N2
D,L-α-Tocopherol acetate	n/a	(2.1)	2.1	–
D,L-α-Tocopherol	n/a	(2.3)	2.3	–
Biotion	n/a	(0.4)	–	–
Retinol, all *trans*	–	(0.3)	0.3	–
Retinol acetate	n/a	(0.2)	0.2	–
BSA	n/a	(37)	37	–
Catalase	n/a	(0.01)	0.01	–
Human recombinant insulin	n/a	(0.6)	0.6	0.8609
Human transferrin (apo-)	n/a	(0.062)	–	–
Holo-transferrin	–	–	0.062	10
Superoxide dismutase	n/a	(0.077)	0.077	–
Corticosterone	n/a	(0.058)	0.058	–
D(+)-Galactose	n/a	(83)	83	–
Ethanolamine	n/a	(16)	16	–
Glutathione (reduced)	n/a	(3.2)	3.2	–
L-Carnitine	n/a	(12)	12	–
Linoleic acid	n/a	(3.5)	3.5	–
Linolenic acid	n/a	(3.5)	3.5	–
Lipoic acid (thioctic acid)	–	(0.2)	0.2	–
Progesterone	n/a	(0.02)	0.02	0.02
Putrescine	n/a	(183)	183	100.06
Selenite	n/a	(0.083)	0.083	0.0301
T3 (triiodo-L-thyronine)	n/a	(0.0026)	0.0026	–

*Note that while the composition of B27 has been published (Brewer et al., 1993), the exact concentrations of its components are not known (indicated by n/a). The table therefore lists the concentrations of the components of B18 in parentheses (Brewer and Cotman, 1989), a precursor to B27, which were retained therein. This may provide a general idea on the comparability of B27 to the other supplements. Likewise, the manufacturer of GS21 states that its formulation is based on the published formulation of NS21 (Chen et al., 2008). The formulation of N2 is available from the manufacturer and corresponds to its original publication (Bottenstein and Sato, 1979). The concentrations shown in the table correspond to the final concentrations in the culture medium.*

**Table 2:** Adapted formulation of BrainPhys medium (Bardy et al., 2015) as used in this study.

BrainPhys component	Concentration (mM)	Company
Sodium chloride	121	Carl Roth
Potassium chloride	4.2	Merck
Calcium chloride	1.1	Merck
Magnesium sulfate	1.0	Sigma
Ferric nitrate	0.000124	Thermo Fisher Scientific
Zinc sulfate	0.0015	Thermo Fisher Scientific
Sodium bicarbonate	26	Sigma
Sodium phosphate dibasic	0.5	Carl Roth
Sodium phosphate monobasic	0.45	Sigma
Glycine	0.002	Carl Roth
L-Alanine	0.002	Carl Roth
L-Serine	0.002	Carl Roth
L-Alanyl-L-glutamine	0.5	Carl Roth
L-Arginine hydrochloride	0.3	Carl Roth
L-Asparagine-H_2_O	0.05	Thermo Fisher Scientific
L-Cysteine hydrochloride-H_2_O	0.1	Carl Roth
L-Histidine hydrochloride-H_2_O	0.15	Carl Roth
L-Isoleucine	0.416	Carl Roth
L-Leucine	0.451	Carl Roth
L-Lysine hydrochloride	0.499	Carl Roth
L-Methionine	0.116	Carl Roth
L-Phenylalanine	0.215	Carl Roth
L-Proline	0.06	Carl Roth
L-Threonine	0.449	Carl Roth
L-Tryptophan	0.0441	Carl Roth
L-Tyrosine disodium salt dihydrate	0.214	Thermo Fisher Scientific
L-Valine	0.452	Carl Roth
D-Glucose	2.5	Sigma
Sodium pyruvate	0.5	Sigma
Choline chloride	0.0641	Thermo Fisher Scientific
D-Calcium pantothenate	0.0047	Thermo Fisher Scientific
Folic acid	0.00601	Sigma
I-Inositol	0.07	Carl Roth
Niacinamide	0.0166	Sigma
Pyridoxine hydrochloride	0.00986	Carl Roth
Thiamine hydrochloride	0.00644	Carl Roth
Cyanocobalamin	0.000502	Carl Roth
Riboflavin	0.000582	Carl Roth
Hepes	5.0	Carl Roth
Phenol red	0.0215	Sigma

### Metabolic Deprivation Experiments

On day 9 of cultivation, neuronal cultures were washed twice with PBS and then incubated under anoxic conditions (0% O_2_, 37°C, 5% CO_2_) in a Concept-400 hypoxia workstation (Ruskinn Technologies) or under normoxic conditions (21% O_2_, 37°C, 5% CO_2_) for 8 h in BSS_0_ (116 mM NaCl, 5.4 mM KCl, 0.8 mM MgSO4, 1 mM NaH_2_PO_4_, 26.2 mM NaHCO_3_, 10 μM glycine, 1.8 mM CaCl_2_, 10 mM HEPES pH 7.4) as described (Mergenthaler et al., 2012). After the experimental procedures, the supernatant was analyzed directly or medium was added to the cells for 24 h until neuronal cell death was quantified by measuring lactate dehydrogenase (LDH) release.

### Lactate Dehydrogenase (LDH) Release Assay

Cell death was evaluated 24 h after metabolic deprivation by measuring LDH release from primary neurons in a coupled spectrophotometric assay as previously described (Niu et al., 2017). Briefly, 50 μl supernatant or 25 μl LDH standard (500 U/l, DiaSys Greiner) for data normalization were mixed with 200 μl of 212 μM β-NADH in 33.3 mM KH_2_PO_4_ and 66.7 mM K_2_HPO_4_ (pH 7.4), and then 25 μl of 22.7 mM pyruvate in 33.3 mM KH_2_PO_4_ and 66.7 mM K_2_HPO_4_ (pH 7.4) to start the reaction. The reduction of β-NADH to NAD+ is proportional to the LDH activity and was measured by absorbance at 340 nm on an MRX revelation (Dynex Technologies) plate reader at room temperature (RT). Total LDH release was measured after incubating neurons with Triton X-100 for 30 min at 37°C (final concentration: 0.5% v/v) and a second measurement with 25 μl supernatant or 25 μl LDH standard was performed. All data were normalized to the total LDH release measurements.

### Lactate Measurements

The colorimetric L-Lactic Acid Assay Kit (AAT Bioquest) was used according to the manufacturer’s instructions to measure lactate levels in the media of rat brain cortical neurons cultured for 9 days. Briefly, 50 μl sample was mixed with 50 μl assay buffer and absorbance was measured after an incubation period of 90 (**Figure 2A**) or 30 min (**Figure 2B**) at 550 nm on an MRX revelation (Dynex Technologies) plate reader at RT. Measurements of each well were normalized to its total protein content as quantified with a BCA Assay Kit (Thermo Fisher Scientific).

### Metabolic Flux Analysis

Metabolic flux was measured using the Seahorse XFe96 Extracellular Flux Analyzer (Seahorse Bioscience, Agilent Technologies). Appropriate cell numbers and compound concentrations were titrated in preliminary experiments (data not shown). The cell seeding density and compound concentrations given below represent conditions with the optimal dynamic range in our preliminary assays. Neurons were seeded at a density of 20,000 cells/well, and cultured for 9 days in NBM or BrainPhys as described above in the presence of the different neuronal supplements (B27, N2, or GS21). Two types of metabolic analyses were performed: the cell mito stress test to assess respiratory activity, and the glycolysis stress test to assess glycolysis (**Figure 3**). Both assays were performed in DMEM-D5030 (Sigma–Aldrich) containing 143 mM sodium chloride and 2 mM L-glutamine (pH 7.35–7.40). The medium was supplemented with 10 mM glucose for the cell mito stress test. Prior to the assays, cultures were washed with the respective medium. To characterize the effects of B27, N2, and GS21 on metabolic flux, neurons were cultured with the respective supplements in the media indicated in the figure legends. The cell mito stress test and glycolysis stress test were performed in the absence or presence of the different supplements. For the latter, the supplements were individually added to the assay medium as indicated in the figure legends so that this incubation was performed in the presence of the same supplement as the cultivation period. Neurons were pre-incubated in this medium for 1 h at 37°C (atmospheric CO_2_) before the start of the measurements. For the cell mito stress test, the sensor cartridge was loaded with 0.75 μM oligomycin (Sigma–Aldrich), 0.75 μM carbonyl cyanide-*p*-trifluoromethoxyphenylhydrazone (FCCP, Sigma–Aldrich), 1 μM rotenone (Sigma–Aldrich), and 1 mM antimycin A (Sigma–Aldrich), and for the glycolysis stress test with 10 mM glucose,1 μM oligomycin, and 100 mM 2-deoxy-D-glucose (2-DG; Carl Roth). These reagents were consecutively injected into each well in this order. Measurements were taken 20 min after pre-incubation and 6, 12, and 18 min after each injection with 3 min mixing intervals in between prior to the next injection. For analysis and depiction in the figures, we used the following measurements after each injection: for the cell mito stress test basal 3, oligomycin 3, FCCP 1, and rotenone/antimycin 2; for the glycolysis stress test basal 3, glucose 3, oligomycin 3, and 2-DG 2. After each experiment, total protein concentration of each well was determined with a BCA Protein Assay Kit (Thermo Fisher Scientific) to normalize oxygen consumption rates (OCR) and extracellular acidification rates (ECAR) measurements to the total protein content of each well.

### Statistics

Neuronal cultures from different embryos were considered as independent observations. Statistical graphing was performed by using GraphPad Prism 5.0. One-way ANOVA and Turkey’s HSD *post hoc* tests were calculated for all experiments with *n* ≥ 3 in GraphPad Prism 5.0 or IBM SPSS Statistics 24.0. A *p* < 0.05 was considered statistically significant. In all figures, this is represented by ^∗^*p* ≤ 0.05, ^∗∗^*p* ≤ 0.01, and ^∗∗∗^*p* ≤ 0.001. Figures display single observations as single data points as well as mean ± SD with a whisker plot.

## Results

### The Neuronal Culture Supplement B27 Protects Neurons from Cell Death Under Glucose Deprivation

**FIGURE 1:**
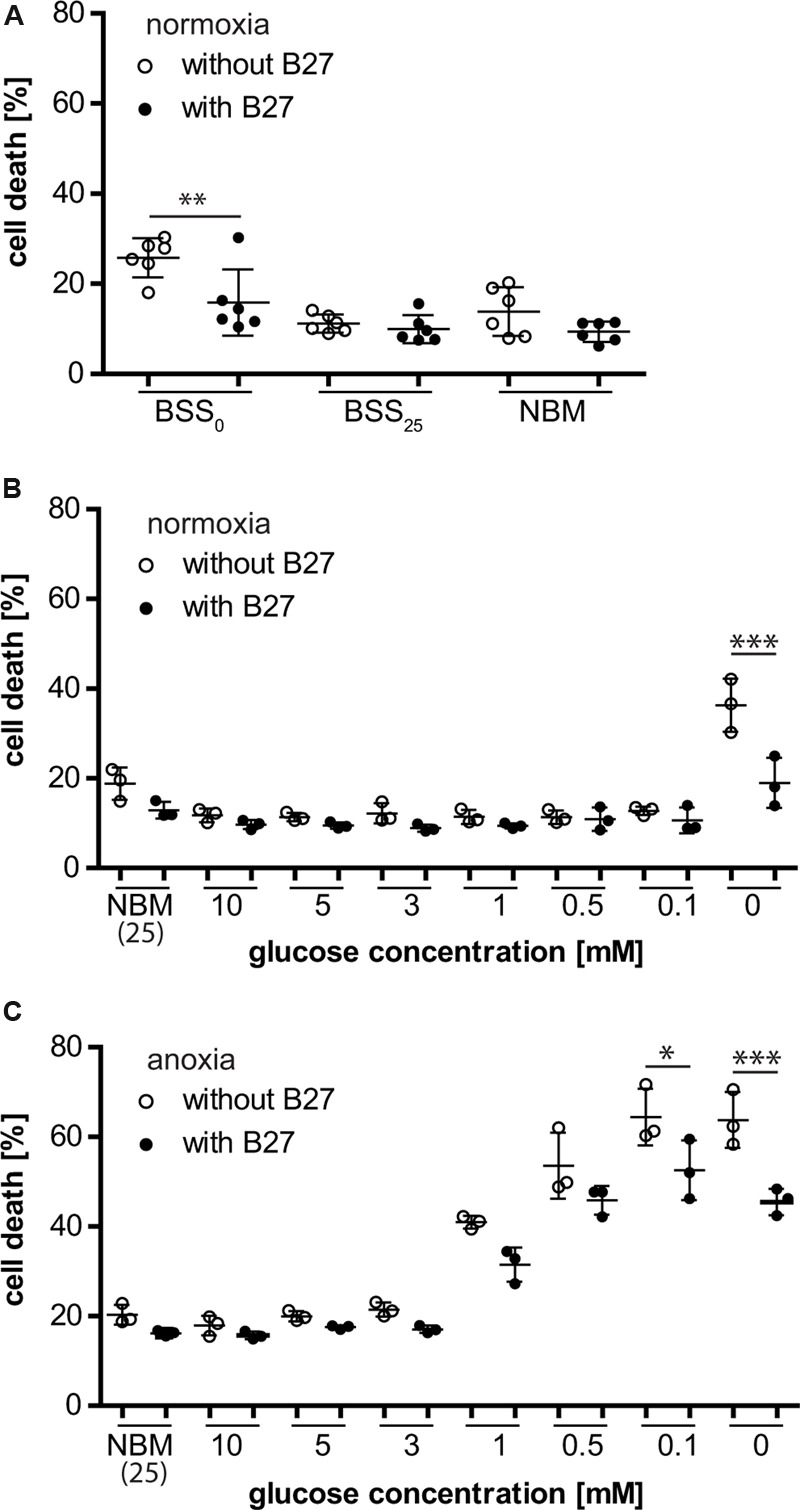
**(A)** B27 treatment for 8 h protected primary neurons undergoing glucose depletion (BSS_0_) for 8 h but did not have an effect on neuronal survival under control conditions (BSS_25_, NBM). **(B)** B27 protected neurons from cell death after glucose depletion for 8 h but not from cell death after glucose deprivation (GD) even at very low glucose concentrations. **(C)** B27 protected neurons from cell death after OGD and oxygen deprivation (OD) at low glucose concentrations.

Neurons that were depleted of glucose (i.e., no glucose present during incubation) for 8 h were rescued from cell death when B27 was added to the deprivation buffer (BSS_0_), indicating that B27 might be able to support neuronal survival for a limited time under these conditions (**Figure 1A**). This effect was not seen when neurons were incubated in NBM or BSS (BSS_25_) containing 25 mM glucose with or without B27 for 8 h. Furthermore, decreasing glucose concentrations, thereby eliciting glucose deprivation (GD), with or without addition of B27 to BSS did not have any effect on neuronal survival under normoxic conditions (**Figure 1B**). However, as before, neurons depleted of glucose were protected from cell death when incubated in the presence of B27. On the contrary, we found that B27 decreased neuronal cell death under oxygen deprivation (OD) at low glucose concentrations, although this effect did not reach statistical significance at 0.5 mM (*p* = 0.49), 1 mM (*p* = 0.197), and higher glucose concentrations (**Figure 1C**). Furthermore, B27 protected neurons from cell death after oxygen–glucose deprivation (OGD), a model of hypoxia–ischemia, when no glucose was present in the deprivation buffer (**Figure 1C**). In summary, these data indicate that B27 is able to protect neurons from cell death triggered by glucose depletion under normoxia and hypoxia, as well as at low glucose levels under hypoxia.

### The Neuronal Culture Supplement B27 Interferes with Glycolysis

**FIGURE 2:**
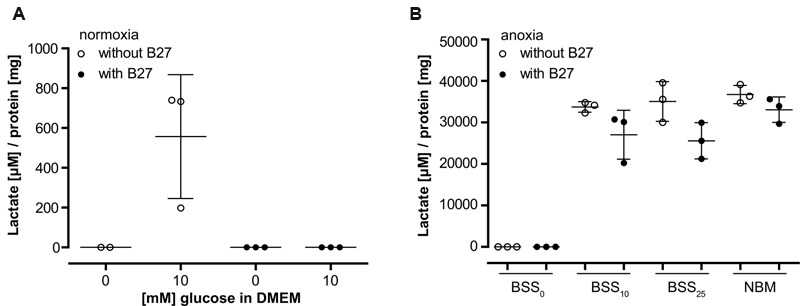
**(A)** Lactate release from neurons was measured after 98 min incubation in DMEM without glucose (see section “Materials and Methods” for metabolic flux analysis) followed by a 15 min incubation with or without addition of 10 mM glucose. Co-incubation with B27 completely abolished lactate release after addition of glucose. **(B)** Lactate release from neurons was measured after 8 h incubation at anoxic conditions using BSS with or without addition of glucose and B27. Note the 30-fold difference in lactate accumulation compared to **(A)**. BSS_0_, BSS_10_, BSS_25_ – 0, 10, and 25 μM glucose in BSS. NBM – 25 μM glucose in NBM.

To further investigate the role of B27 in protecting neurons from cell death under metabolic deprivation, we incubated cultured neurons in the basal medium DMEM-D5030 for 98 min without glucose as described in the section “Materials and Methods” for metabolic flux analysis. As expected, addition of 10 mM glucose resulted in glucose turnover and accumulation of lactate over a period of 15 min (**Figure 2A**). However, consistent with a profound effect on neuronal glucose metabolism, addition of B27 together with 10 mM glucose completely abolished lactate accumulation in that timeframe (**Figure 2A**). Next, we added B27 to neurons undergoing hypoxia with different glucose concentrations in BSS or NBM containing 25 mM glucose (**Figure 2B**). Under all conditions, lactate accumulation was decreased by the addition of B27, although this effect did not reach statistical significance (BSS_10_ *p* = 0.295, BSS_25_ *p* = 0.055, and NBM *p* = 0.881). Together, these data suggest that B27 interferes with glycolysis.

### Metabolic Flux Analysis Allows Investigating the Impact of the Culture Microenvironment on Neuronal Metabolic Function

**FIGURE 3:**
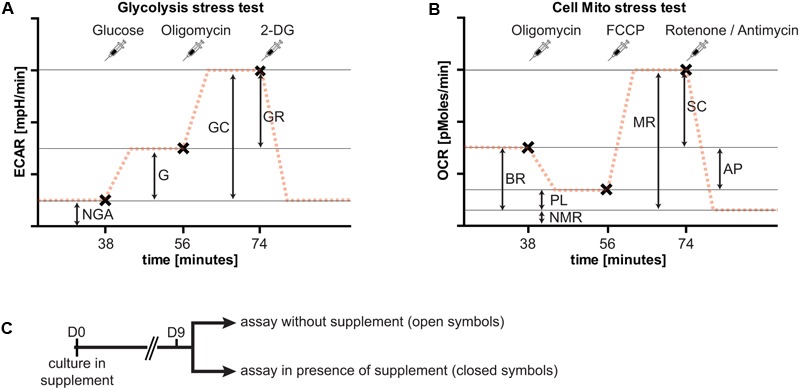
Overview of the experimental paradigm of metabolic flux analysis. Importantly, extracellular acidification rates (ECAR) and oxygen consumption rates (OCR) are always measured at the same time. For clarity, only one of the two measurements is shown here for each assay. **(A)** The glycolysis stress test allows determination of non-glycolytic acidification (NGA), glycolysis (G), glycolytic capacity (GC), and glycolytic reserve (GR). At specified intervals (syringes, x), glucose to start glycolytic flux (38 min after start of the assay), oligomycin to block respiration (after 56 min), and 2-deoxy-D-glucose (2-DG, after 74 min) to block glycolysis are added. The addition of glucose results in lactate release-dependent increase in extracellular pH, characterized by the ECAR, and a further increase by blockade of oxidative phosphorylation. Addition of 2-DG inhibits glycolysis and decreases extracellular acidification to basal rates. **(B)** The cell mito stress test allows determination of basal respiration (BR), proton leak (PL), non-mitochondrial respiration (NMR), maximal respiration (MR), respiratory spare capacity (SC), and ATP production (AP). At specified intervals (syringes, x), oligomycin to block basal respiration by inhibiting complex V leading to decreased OCR (after 38 min), carbonyl cyanide-*p*-trifluoromethoxyphenylhydrazone (FCCP) to uncouple mitochondria resulting in MR (after 56 min), and rotenone and antimycin A to block complex I and III, respectively, of the respiratory chain, thereby completely inhibiting mitochondrial respiration (after 74 min) are added. **(C)** Overview of the culture paradigm used for the experiments described in **Figures 4 and 5**. Neurons are cultured in the respective supplement for 9 days. On the day of metabolic flux analysis, the medium was changed to assay medium either with or without the supplement used for culturing neurons (see section “Materials and Methods” for details).

**FIGURE 4:**
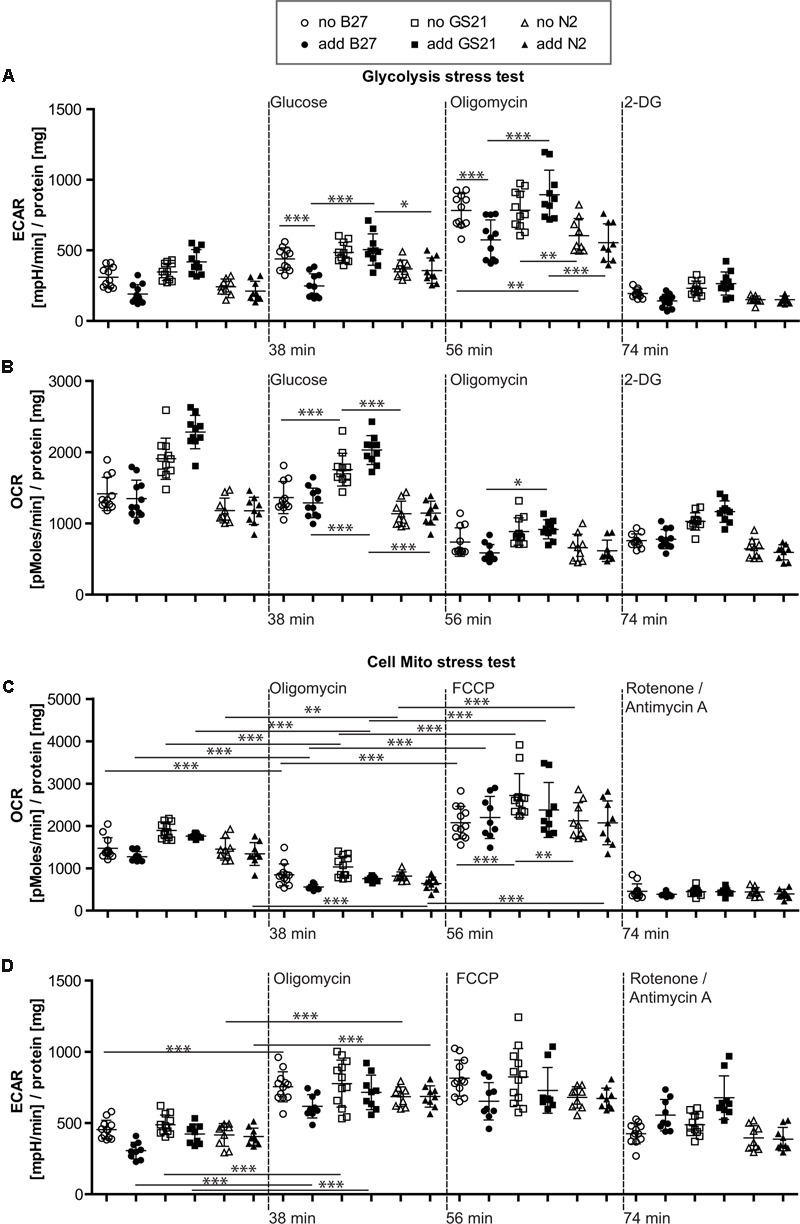
**(A,B)** Glycolysis stress test after cultivation of neurons in Neurobasal medium. **(A)** Addition of B27 acutely inhibited glycolytic pathways by decreasing glycolysis as well as the glycolytic capacity (circles), while addition of N2 (triangles) did not further inhibit metabolic function. Compared to GS21 (squares), glycolytic capacity of neurons was substantially decreased with B27 or N2. **(B)** Respiratory activity was increased after cultivation with GS21 and after its addition to neurons undergoing metabolic flux analysis compared to cultivation with or addition of B27 or N2. **(C,D)** Cell mito stress test after cultivation in Neurobasal medium. **(C)** Maximal respiration was highest after cultivation with GS21 but measurements were performed in the absence of the supplement (open squares). **(D)** Glycolysis was not affected under these conditions. Open symbols indicate that neurons were cultured in the presence of the respective supplement but that was not present during measurements. Solid symbols indicate that it was also present during measurements. The time points of addition of the injection of the respective reagents after start of the metabolic flux assay are indicated with dotted lines.

**FIGURE 5:**
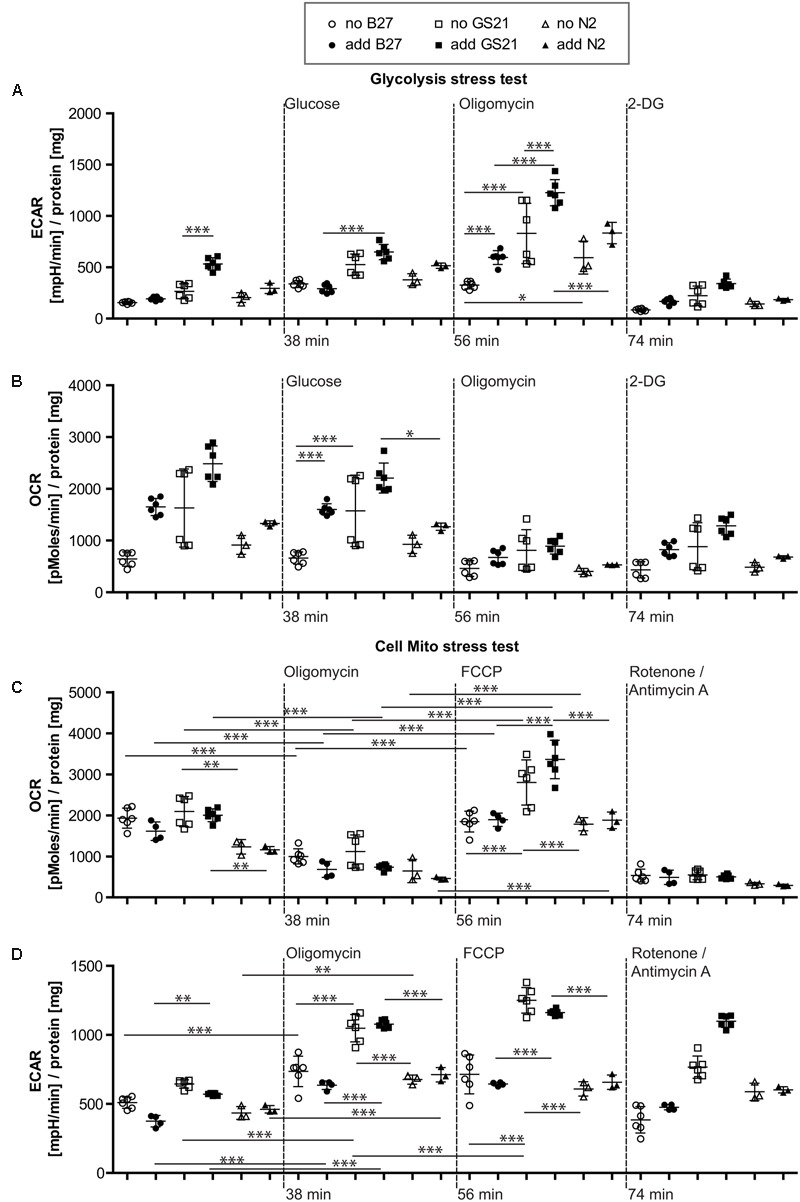
**(A,B)** Glycolysis stress test after cultivation of neurons in BrainPhys medium. **(A)** Glycolysis in the presence of GS21 (solid squares) was significantly higher than in the presence of B27 (solid circles). Glycolytic capacity was significantly higher with (solid squares) than without (open squares) GS21 and also compared to B27 (circles) and N2 (triangles) for each condition. **(B)** Respiratory activity was stimulated by addition of B27 (circles) and higher with GS21 (solid squares) than with N2 (sold triangles). **(C,D)** Cell mito stress test after cultivation in BrainPhys medium. **(C)** Basal respiration was higher with GS21 (squares) than with N2 (triangles) and respiratory capacity with GS21 (squares) higher than with B27 (circles) or N2 (triangles). **(D)** Glycolysis was higher after respiratory inhibition with oligomycin and after mitochondrial uncoupling with FCCP after cultivation with GS21 (squares) compared to the other supplements. After rotenone/antimycin A treatment, only cultures exposed to GS21 (squares) retained active glycolysis. Open symbols indicate that neurons were cultured in the presence of the respective supplement but that was not present during measurements. Solid symbols indicate that it was also present during measurements. The time points of addition of the injection of the respective reagents after start of the metabolic flux assay are indicated with dotted lines.

To further investigate the metabolic changes elicited by neuronal culture conditions, we performed live cell metabolic flux analysis (**Figure 3**). This assay allows simultaneous measurements of the two fundamental pathways of glucose utilization by measuring ECAR (**Figure 3A**) as a measure of neuronal lactate production, and OCR (**Figure 3B**) as an indicator of neuronal respiratory chain activity. Addition of substrates and inhibitors in sequential order as indicated allows dynamic measurements of metabolic parameters of glycolysis and oxidative phosphorylation (see **Figure 3** and “Materials and Methods” for details). In the following, neurons were cultured in the presence of B27 or the alternative neuronal culture supplements N2 and GS21, and we performed metabolic flux measurements in the presence (closed symbols in **Figures 4, 5**) or absence (open symbols in **Figures 4, 5**) of the respective culture supplement (**Figure 3C**).

### The Neuronal Culture Microenvironment Can Mediate Opposing Function on Neuronal Glucose Metabolism

When directly assaying glycolysis, we found that addition of B27 to the assay medium acutely inhibited glycolytic pathways by decreasing glycolysis as well as the glycolytic capacity (**Figure 4A**, circles) but did not change respiratory activity (**Figure 4B**, circles). When using the N2 supplement instead of B27 (**Figures 4A,B**, triangles), we also measured inhibition of metabolic function. Compared to the more modern neuronal culture supplement GS21 (squares), addition of B27 or N2 substantially decreased glycolytic capacity of neurons (**Figure 4A**). In addition, respiratory activity (OCR, **Figure 4B**) was increased after cultivation (open squares) with GS21 and after its addition (solid squares) to neurons undergoing metabolic flux analysis compared to cultivation with or addition of B27 (circles) or N2 (triangles). When we directly probed neuronal respiratory activity (**Figures 4C,D**), we found that maximal respiration was highest when neurons were cultured in the presence of GS21 but measurements were performed in the absence of the supplement (**Figure 4C**, open squares). Glycolysis was not affected under these experimental conditions (**Figure 4D**). Thus, our data indicate that the crucial supplements for culturing neurons may alter metabolic function immediately after their addition to the assay medium as well as when they are continuously present in the culture media. However, this effect is complex, as no single ingredient of B27 (**Table 1**) had a comparable effect on neuronal metabolism (Supplementary Figure 1). Importantly, culturing neurons in the presence of GS21 resulted in a denser neurite network compared to B27 or N2 (Supplementary Figure 2), thereby corroborating the results by Chen et al. (2008) that B27 can negatively affect neuronal synaptic plasticity and neurite integrity.

Finally, we investigated the metabolic function of neurons cultivated in the recently introduced BrainPhys medium (**Figure 5**), which is considered to be more physiological than previous neuronal culture media. In contrast to NBM (**Figure 4**), B27 and N2 did not inhibit glycolysis when using BrainPhys medium and the supplements were added for the measurements. However, adding GS21 during measurements still resulted in significantly higher glycolysis compared to B27 and significantly higher glycolytic capacity compared to B27 and N2 (**Figures 5A,B**). Furthermore, mitochondrial respiratory function analyzed by measuring maximal respiration was significantly higher when neurons were incubated with GS21 during OCR measurements. Finally, ATP-producing glycolysis after complete inhibition of electron transport with rotenone and antimycin A (increased ECAR, **Figure 5D**) only remained active under culture and treatment conditions using GS21.

## Discussion

Here, we describe the profound effect of the neuronal culture microenvironment on neuronal energy metabolism and neuronal survival under metabolic deprivation. We demonstrate that long-standing protocols and culture paradigms for culturing rodent primary neurons (Bottenstein and Sato, 1979; Brewer et al., 1993) have profound effects on neuronal metabolic function. To the same end, our data suggest that different culture conditions as well as acute stimulation with neuronal culture supplements may fundamentally determine the preferential use of bioenergetic pathways in neurons.

**FIGURE 6:**
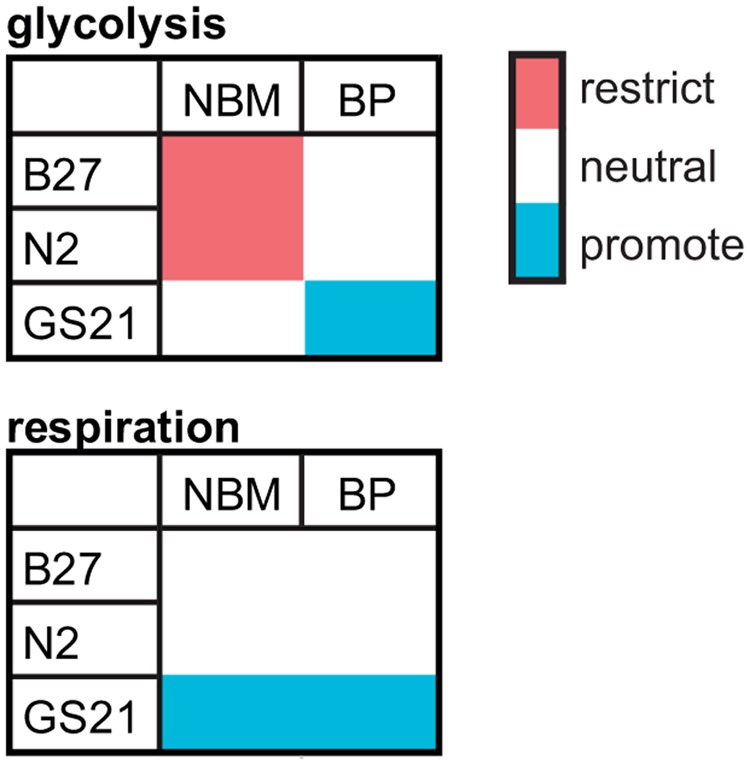
Qualitative summary of the effect of B27, N2, and GS21 in combination with NBM or BrainPhys (BP) on the main metabolic pathways analyzed by metabolic flux analysis in this study.

Specifically, we find that the commonly used neuronal cell culture supplement B27 can protect primary neurons from cell death after glucose depletion as well as OD under low glucose conditions and OGD. However, we find that B27 as well as the alternative neuronal supplements N2 and GS21 have different effects on neuronal energy metabolism (**Figure 6**). In that regard, B27 restricts glycolysis, an effect also seen with N2 but not with GS21. In addition, neurons cultured with GS21 had the highest maximal mitochondrial respiration, which represents the highest activity in oxidative phosphorylation neurons can achieve. Importantly, oxidative phosphorylation is the major mechanism powering neuronal activity (Hall et al., 2012). Finally, we find a complex interplay exists between neuronal culture supplements and the culture medium. When using BrainPhys medium instead of NBM, B27 and N2 did not inhibit glycolysis; however, GS21 still resulted in higher glycolytic and respiratory rates. One of the components that may be involved in mediating this effect is lipoic acid, which is one of the components of NS21 (Chen et al., 2008), the published formulation of GS21. Neither B27 nor N2 contains lipoic acid. However, the cofactor for α-ketoacid dehydrogenases, which play an important role in mitochondrial energy metabolism (Shay et al., 2009), had been present in B18 (Brewer and Cotman, 1989), the precursor to B27. Importantly, administration of lipoic acid, albeit at significantly higher concentrations than present in NS21, has been suggested to modulate glucose uptake and metabolism in the brain *in vivo* and in neurons *in vitro* through Akt/JNK signaling (Jiang et al., 2013).

*In vitro* systems play a crucial role for disease modeling in general and for neurobiology in particular (Mergenthaler et al., 2012; Bosche et al., 2013; Hermann et al., 2015). Therefore, defined media have been developed to support growth and maintenance of different cell types of the brain, and historically culture conditions have been optimized with regards to cellular viability, matching gene expression to *in vivo* conditions, or replicating *in vivo* phenotypes (Livesey, 2015). However, our data highlight that in addition to considering neuronal activity, metabolic parameters require equal attention in neuronal culture models.

Although controversies exist over the cell types contributing to oxidative or glycolytic glucose consumption in the brain, neuronal computation and synaptic transmission are tightly coupled to neuronal energy metabolism (Mergenthaler et al., 2013). Furthermore, synaptic activity, which accounts for most of the brain’s energy expenditure (Harris et al., 2012; Mergenthaler et al., 2013), has been suggested to genetically reprogram neuronal energy metabolism (Bas-Orth et al., 2017) further supporting the close link between neuronal energy use and computation. It is therefore not surprising that in addition to affecting neuronal metabolic function (**Figures 4, 5**), different neuronal culture supplements and culture media also affect synapse formation, neurophysiological function, as well as neuronal viability (Chen et al., 2008; Cressey, 2009; Bardy et al., 2015). In that regard, NBM was recently shown to suppress synaptic activity (Bardy et al., 2015). Furthermore, it has been suggested that NBM may trigger excitotoxicity under certain conditions as it contains high concentrations of L-cysteine that may activate NMDA receptors (Hogins et al., 2011). Although the classical neuronal culture supplements N2 or B27 were not shown to acutely affect neuronal electrical activity when used together with BrainPhys (Bardy et al., 2015), our data suggest a significant effect on the metabolic activity of neurons elicited by these culture supplements (**Figure 5**). Furthermore, since the exact composition of B27 is kept proprietary (Cressey, 2009) despite its publication (Brewer et al., 1993), its use may be limited for certain areas of neuroscience research, such as in the field of neuroendocrinology (Roth et al., 2010).

The advent of stem cell technology to generate human neurons (Brennand et al., 2015) has boosted cell culture-based research of brain function as well as human disease modeling (Sandoe and Eggan, 2013). However, despite profound methodological advances to investigate specific metabolic pathways to (neuronal) homeostasis, physiological modeling of the neuronal microenvironment is rarely considered in current modeling concepts (Livesey, 2015). To that end, powerful experimental tools enable investigating metabolic function in brain cells on a molecular (Hung et al., 2011; Tantama et al., 2013) and cellular (San Martin et al., 2014; Lundgaard et al., 2015) level and even allow probing individual metabolites (Yaseen et al., 2013) in a functional context. Bioenergetic profiling using extracellular metabolic flux analysis (Dranka et al., 2011) provides a novel potent tool for dissecting the contribution of oxidative phosphorylation and glycolysis to neuronal function. Extracellular metabolic flux analysis measures oxygen consumption or extracellular acidification in the culture medium over time, thereby providing surrogate parameters for mitochondrial respiration or glycolytic lactate release. However, given the nature of these analytes, it is important to keep in mind that changes therein can stem from other sources than altered mitochondrial respiration or glycolysis. For example, multifactorial formation of CO_2_ or conditions where pyruvate oxidation is altered can result in ECAR changes that are not a consequence of changes in the glycolytic rate (Divakaruni et al., 2014).

Imaging techniques such as positron emission tomography to investigate metabolic function in the human and rodent brain under physiological (Vaishnavi et al., 2010; Aanerud et al., 2012) and pathophysiological (Vlassenko et al., 2010; Catana et al., 2012; Heiss, 2014; Stender et al., 2015) conditions have come of age. Despite central contributions from human functional brain imaging as well as a large variety of experimental systems to understanding brain function, controversies on fundamental aspects of brain metabolism and the contribution of different cell types in the brain to metabolic function remain (DiNuzzo et al., 2010; Jolivet et al., 2010; Mangia et al., 2011; Dienel, 2012a,b; Pellerin and Magistretti, 2012; Mergenthaler et al., 2013; Lundgaard et al., 2015; Magistretti and Allaman, 2015; Machler et al., 2016), further highlighting the need for sound cellular modeling of brain function.

## Conclusion

Together with the development of novel culture systems for rodent and human neurons (Chen et al., 2008; Bardy et al., 2015), our data provide an important foundation for future studies investigating the contribution of bioenergetic maintenance to physiological brain function or the role of deranged metabolic pathways in neurodegeneration. Investigating metabolic flux in neurons and a vast variety of other cell types has become an important tool in investigating neuronal/cellular function. Ultimately, our data support future developments in neuronal cell culture techniques and point out that careful control of the metabolic environment is an essential component in modeling brain function and the cellular and molecular pathophysiology of brain disease in culture.

## Ethics Statement

This study was carried out in accordance with the recommendations of the Landesamt für Gesundheit und Soziales (LAGeSo), Berlin. The protocol was approved by the Landesamt für Gesundheit und Soziales (LAGeSo) Berlin.

## Author Contributions

JS performed experimental work, collected and analyzed the data, and compiled draft versions of this manuscript. BB discussed the data, and critically revised data analyses and drafts of this manuscript. AM allocated funding support to this project, discussed the data and analyses, and critically revised drafts of this manuscript. PM conceived and supervised all aspects of this work, analyzed and discussed the data, and wrote the paper. All authors have read and approved the final version of the manuscript.

## Conflict of Interest Statement

BB received speaker honoraria, travel and material support from CSL Behring, Germany/Canada. BB is member of the scientific advisory board of Edge Therapeutics Inc. The other authors declare that the research was conducted in the absence of any commercial or financial relationships that could be construed as a potential conflict of interest.
