# Supplementary material for: Neuronal Culture Microenvironments Determine Preferences in Bioenergetic Pathway Use
Source: Front Mol Neurosci. 2017 Sep 29;10:305. doi: 10.3389/fnmol.2017.00305 (PMC5649214; doi:10.3389/fnmol.2017.00305)
Supplement: Supplementary file 1 [file Data_Sheet_1.pdf]

Supplemental Material to

Sünwoldt J, Bosche B, Meisel A and Mergenthaler P (2017)
Neuronal Culture Microenvironments Determine Preferences in Bioenergetic Pathway Use.
Front. Mol. Neurosci. 10:305.; doi: 10.3389/fnmol.2017.00305

Supplemental material – Figure 1

Here, the glycolysis stress test was performed after cultivation of neurons in Neurobasal media in the presence of commercial B27. For the assay, neurons were incubated with the individual component shown supplemented to the assay medium. In conclusion, individual components of B27 do not show a comparable effect on metabolic flux as the commercial B27 mix. As before, both ECAR and OCR measurements are given. See the table for each component and the concentration used. For details on the assay, see the methods section of the manuscript.

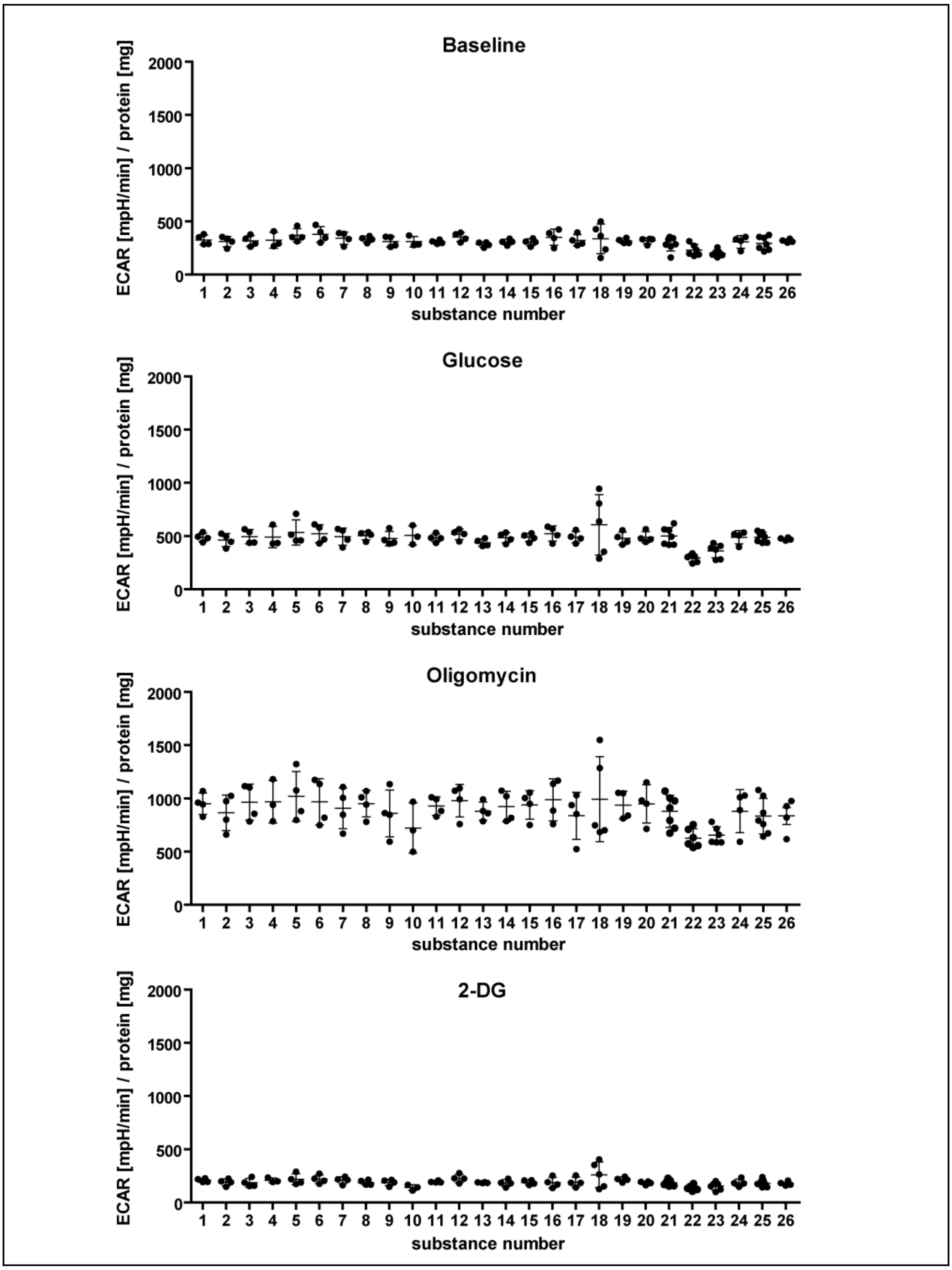

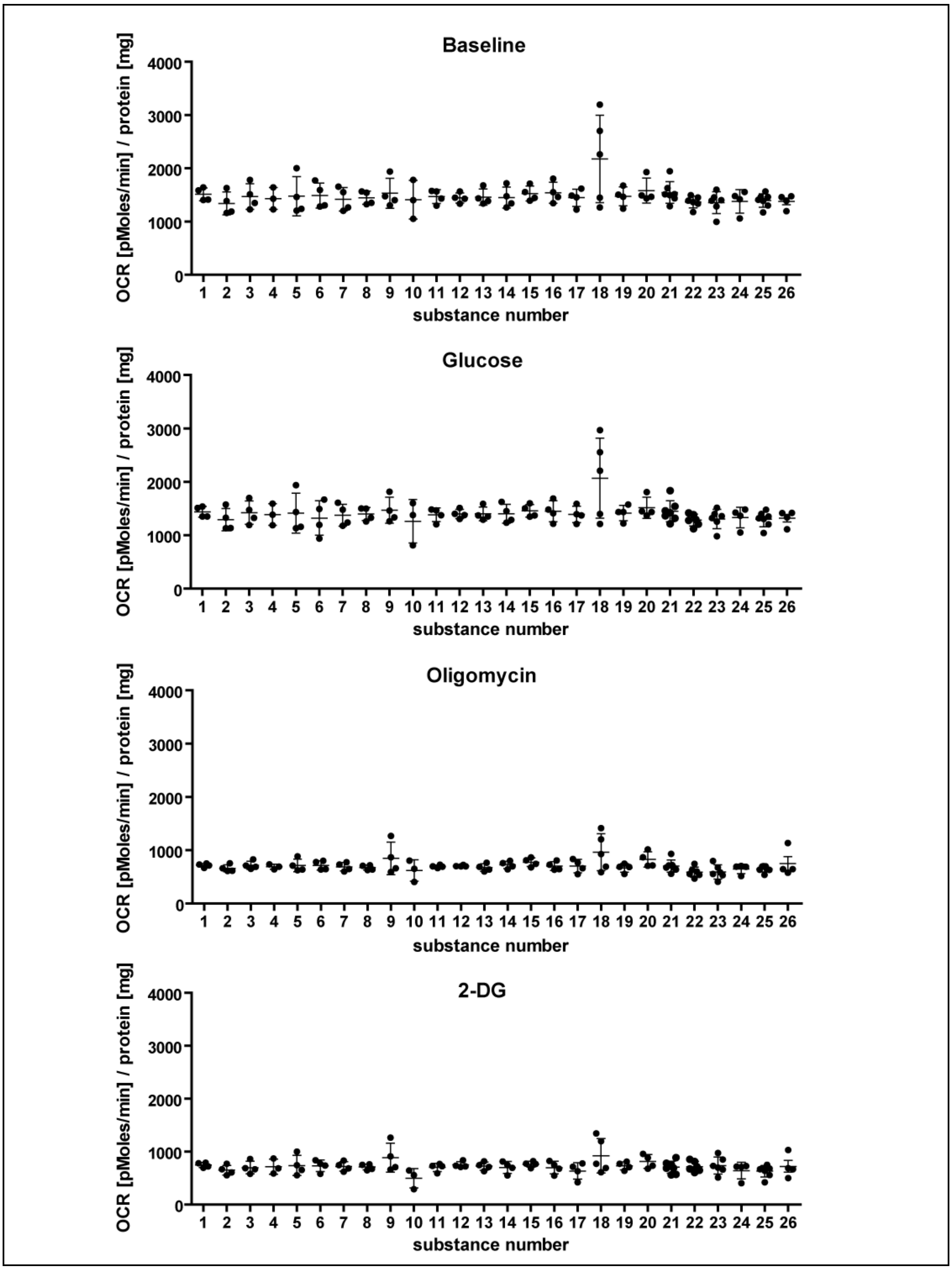
Baseline
Glucose
Oligomycin
2-DG
OCR [pMoles/min] / protein [mg]
0
1000
2000
3000
4000
1 2 3 4 5 6 7 8 9 10 11 12 13 14 15 16 17 18 19 20 21 22 23 24 25 26
substance number

Number	Component	Concentration	Solvent
1	Progesterone	0.0063 µg/ml	Ethanol
2	Linolenic Acid	1 µg/ml	Ethanol
3	Linoleic Acid	1 µg/ml	Ethanol
4	DL Alpha Tocopherol	1 µg/ml	Ethanol
5	DL Alpha Tocopherol acetate	1 µg/ml	Ethanol
6	Retinyl Acetate	0.1 µg/ml	Ethanol
7	Corticosterone	0.02 µg/ml	Ethanol
8	Human Recombinant Insulin	4 µg/ml	Ready to use
9	Human Transferrin	5 µg/ml	Aqua dest.
10	Superoxide Dismutase	2.5 µg/ml	Aqua dest.
11	Sodium Selenite	0.01435 µg/ml	Aqua dest.
12	D-Galactose	15 µg/ml	Aqua dest.
13	Ethanolamine HCl	1 µg/ml	Aqua dest.
14	Biotin	0.1 µg/ml	Aqua dest.
15	T3 (triodo-I-thyronine)	0.002 µg/ml	Sodium hydroxide
16	Putrescine 2HCl	16.1 µg/ml	Aqua dest.
17	Glutathione (reduced)	1 µg/ml	Aqua dest.
18	BSA, fatty acid free Fraction V	2500 µg/ml	Assay medium
19	Catalase	2.5 µg/ml	Aqua dest.
20	L-Carnitine HCl	2 µg/ml	Aqua dest.
21	Without B27	/	/
22	B27 (commercial)	/	/
23	B27 (self-made)	/	/
24	Ethanol (100%)	0.01 µl/ml	/
25	Aqua dest.	2.5 µl/ml	/
26	Sodium Hydroxide	0.08 µg/ml	/

Concentrations of B27 components

Substances were dissolved in aqua dest., ethanol or sodium hydroxide. Assay medium = DMEM D5030 (Sigma).

Supplemental material – Figure 2

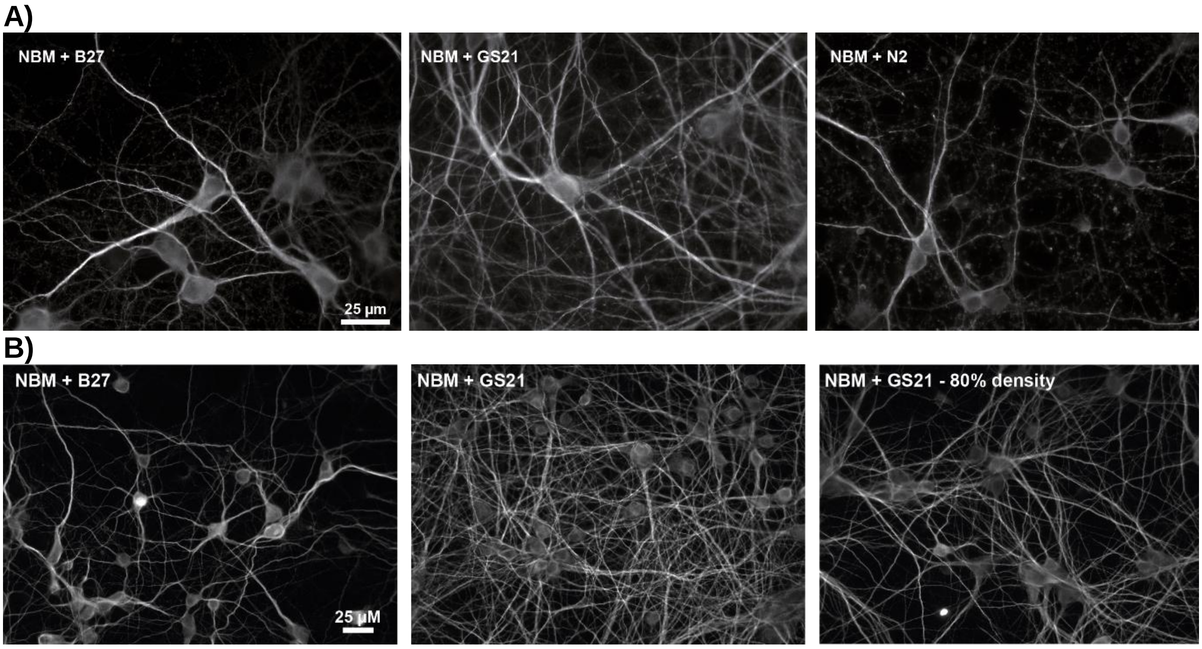

Neurons were fixed and stained for the neuronal marker Map2 after cultivation. **(A)** Primary rat cortical neurons were cultured in Neurobasal media for 9 days in the presence of either B27, GS21 or N2 supplement. **(B)** The density of the neuronal network of GS21-cultivated neurons is higher, even when the cell number at seeding was reduced to 80% of the cell number of B27-cultured neurons.

Methods for immunofluorescence

Primary cortical neurons were washed twice with PBS, cells were fixed with 4% paraformaldehyde for ten minutes at room temperature and washed three times with PBS for five minutes and then incubated with saponine in PBS (final concentration: 0.5%) for 20 minutes at room temperature. Cortical neurons were washed again with PBS containing 0.1% saponine and incubated with a mouse-anti-MAP2 (Sigma) antibody in PBS + 0.1% saponine for one hour at room temperature. Neurons were washed three times with 0.1% saponine in PBS, and incubated with a goat-anti-mouse-Alexa Fluor546 (Thermofisher Scientific) antibody in PBS + 0.1% saponine for one hour at room temperature. Images were taken on a Leica DMI 6000 epifluorescence microscope equipped with a DFC360FX CCD camera and a HCX PL APO 63x NA1.30 objective.
